# Solitary fibrous tumour presenting with a single bone metastasis: report of six cases and literature review

**DOI:** 10.1186/s13569-016-0055-1

**Published:** 2016-09-01

**Authors:** Vittoria Colia, Salvatore Provenzano, Carlo Morosi, Paola Collini, Salvatore Lorenzo Renne, Paolo G. Dagrada, Claudia Sangalli, Angelo Paolo Dei Tos, Andrea Marrari, Paolo G. Casali, Silvia Stacchiotti

**Affiliations:** 1 Adult Mesenchymal Tumour & Rare Cancer Medical Oncology Unit, Cancer Medicine Department, Fondazione IRCCS Istituto Nazionale Tumori, via G. Venezian, 1, 20133 Milan, Italy; 2 Department of Radiology and Radiotherapy, Fondazione IRCCS Istituto Nazionale Tumori, Milan, Italy; 3 Department of Diagnostic Pathology and Laboratory Medicine, Fondazione IRCCS Istituto Nazionale dei Tumori, Milan, Italy; 4 Laboratory of Experimental Molecular Pathology, Department of Diagnostic Pathology and Laboratory Medicine, Fondazione IRCCS Istituto Nazionale dei Tumori, Milan, Italy; 5 Department of Diagnostic Pathology, General Hospital, Treviso, Italy; 6 Humanitas Cancer Center, Milan, Italy

**Keywords:** Sarcoma, Solitary fibrous tumour, Hemangiopericytoma, Metastasis, Prognosis, Bone

## Abstract

**Background:**

Solitary fibrous tumour (SFT) is a rare soft tissue sarcoma with a low metastatic potential. A higher metastatic rate is observed in the high-grade/dedifferentiated variant. The most common expected site of distant spread are the lungs and the liver. Bone involvement is generally viewed as a late stage of disease spread. We report on a retrospective series of SFT patients relapsing with a single distant bone recurrence as first metastatic event, without evidence of other organ involvement.

**Case presentation:**

All patients affected by a single distant bone metastasis from SFT as first distant event, without any evidence of other site of metastasis, observed at our Institution, were considered. Bone involvement from SFT was pathologically assessed in all cases and confirmed by expert pathologists. A total of six patients were retrospectively identified. Primary tumour arose from the meninges in four patients, from soft tissues in two. Bone metastases were located to the vertebrae, the hip, the acetabulum and the rib. In all cases, bone relapse was the first event, with one patient presenting a local relapse. Median time from the primary tumour and the evidence of bone relapse was 40 months (range 0–58). In 2/6 patients bone metastasis was treated with radiotherapy (RT), in 2/6 with surgery, in 2/6 with surgery plus RT. At a median follow-up of 55 months (range 23–88), 5/6 patients are alive (2/5 without disease, 3/5 with multicentric metastatic disease) and one is dead of disease. 2/6 patients did not relapse after the treatment of the bone metastasis.

**Conclusions:**

This small series in a relatively rare histology suggests that isolated, possibly late, bone metastases are a plausible scenario, in particular in meningeal SFT. Notably, new bone lesions in a patient with a history of SFT should be always investigated. Exclusive local treatments may be an option, though collection of such series would be needed to define the best treatment strategy.

## Background

Solitary fibrous tumour (SFT) is a very rare sarcoma, most frequently occurring in middle-aged patients. SFT can occur in several anatomic sites like meninges, peritoneum, head and neck, extremities, and viscera [1–3]. Recently also primary SFTs arising from the bone have been reported [4]. SFT is characterized by a specific *NAB2*–*STAT6* gene fusion which is responsible for the nuclear expression of the chimeric oncoprotein STAT6, which is the immunohistochemical hallmark of SFT [5–8] and helps in differential diagnosis. Of note, dedifferentiated SFT may lose the protein expression while retaining the fusion gene [9]. SFTs are known for the low tendency of recurrence and the low metastatic potential after complete resection (10–15 %), even if a higher metastatic rate (40 %) has been described in case of pleomorphic/dedifferentiated SFT [10, 11]. Recurrence may happen many years after the initial diagnosis [12]. As for all other sarcomas, the most frequent and initial site of metastasis is the lung, followed by the liver [13]. Bone involvement is reported in the late phase of the disease, in patients already affected by lung lesions [12].

We report on a retrospective series of SFT patients who suffered from a single distant bone recurrence as their first metastatic event, without evidence of any other organ involvement.

## Case presentation

From May 2014 to April 2016 at the Fondazione IRCCS Istituto Nazionale Tumori Milan, Italy, we observed five patients with a diagnosis of SFT relapsed with single bone metastasis plus an additional case whose bone metastasis was synchronous to the primary tumour.

Bone involvement from SFT was pathologically assessed in all cases and final diagnosis of bone relapse from SFT was confirmed by expert pathologists basing on morphologic and immunohistochemical features, with STAT6 nuclear immunopositivity, and by comparing the metastatic tissue with the primary tumour.

**Fig. 1:**
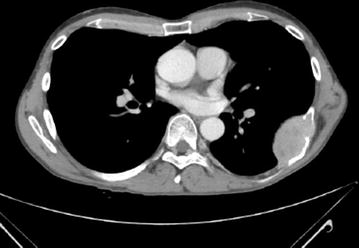
Single bone metastasis from meningeal SFT (patient 1 in Table 1): CT scan (venous phase after contrast medium) shows a solid lesion characterized by homogeneous contrast enhancement at the level of the seventh left rib

Disease status was assessed in all the patients by whole body CT scan, MRI and/or CT of the primary tumour site. A bone scan ruled out the presence of other metastatic bone lesions (Fig. 1).

**Fig. 2:**
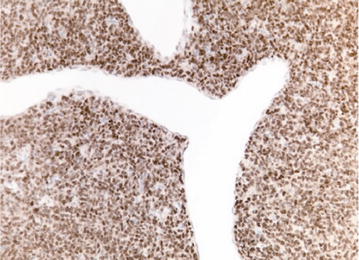
Histopathological pattern of primary meningeal malignant SFT (patient 3 Table 1). Tumour shows patternless growth of a uniform, bland, hypercellular, STAT6 positive, spindle cell proliferation around characteristic thin walled branched vessel. *STAT6 200×*

**Fig. 3:**
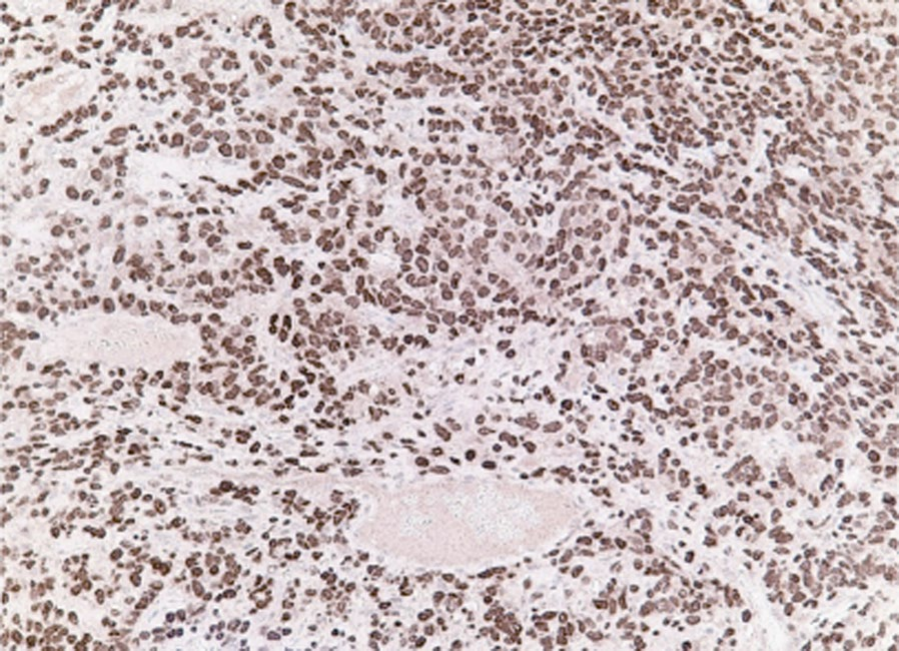
Histopathological pattern of hipbone metastasis from primary meningeal malignant SFT (patient 3 Table 1). Similarly to primary lesion, tumour growths with patternless architecture, around thin walled vessel, with a more loose stroma, retaining immunoreactivity for STAT6. *STAT6 200×*

Patient characteristics are summarized in Table 1. Primary SFT arose from the meninges in four patients, while in the soft tissues of the left thigh and left gluteus in two. Pathological centralized review of the primary tumour confirmed a diagnosis of malignant SFT in all the cases but one that was consistent with a classic SFT. The bone lesions were all consistent with a diagnosis of malignant SFT, with evidence of progression from a classic SFT towards a malignant SFT in one (Figs. 2,3).

**Table 1:** Patient clinical characteristics and tumour histopathological and immunohistochemical features

Case no	Age/sex	Primary tumour					Bone metastasis					Further relapse		Status at last follow-up	OS (months)
		Site	Diagnosis	STAT6 (IHC)	Surgery	RT	Fist distant relapse	Path diagnosis	IHC STAT6	Time from primary and bone relapse (month)	Treatment	Site of relapse	Time to relapse from bone met		
1	38/M	Meninges	Malignant SFT	Pos	Yes	No	7° left rib	Malignant SFT	Pos	50	Complete surgery	Bone	84	AWD	88
2	40/M	Meninges	Malignant SFT	Pos*	Yes	Yes	S3–S4 vertebra	Malignant SFT	Pos	27	palliative RT	Bone and lung	30	AWD	35
3	24/M	Meninges	Malignant SFT	Pos*	Yes	Yes	Hipbone	Malignant SFT	Pos	58	Complete surgery and RT	Bone	78	AWD	79
4	26/F	Meninges	Malignant SFT	Pos	Yes	Yes	C4–C5 vertebrae	Malignant SFT	Pos	48	Complete surgery and RT	NA	NA	NED	51
5	71/F	Deep soft tissue of left thigh	Malignant SFT	Pos	Yes	No	Left acetabulum	Malignant SFT	Pos	54	Definitive RT	NA	NA	NED	56
6	66/M	Deep soft tissue of left gluteus	Classic SFT	Pos	Yes	No	4° right rib	Malignant SFT	Pos*	0	Complete surgery	Lung	12	DOD	23

Bone metastases were mainly detected by the clinical complaint of pain, since a bone scan was not foreseen in the follow-up plan of these patients. Median time from the primary tumour diagnosis and the evidence of bone relapse was 40 months (range 0–58). In five cases, bone relapse was the first event while one patient presented with a synchronous single bone lesion (case 6 in Table 1).

All the patients received a definitive treatment of the bone lesion, with curative intent. A complete surgical resection of the bone metastasis was performed in four cases, followed by complementary radiotherapy in two cases. Radiotherapy was given in two cases.

At a median follow-up of 55 months (range 23–88), five of six patients are alive (2/5 without disease, 3/5 with multicentric metastatic disease) and one is dead of disease. Two of six patients (one treated with definitive RT and one with surgery plus RT) did not suffer of any tumour relapse after the treatment of the bone metastasis, at a follow-up of 51 and 56 months (Table 1).

## Discussion

This retrospective analysis reports on a series of six patients affected by a single solitary bone metastasis from SFT as first metastatic event. This small series in a relatively rare histology shows that isolated, possibly late, bone metastases are a plausible scenario, in particular in meningeal SFTs.

All patients were treated with a curative intent. Two of them are still disease free at 51 and 56 months.

In the literature, the most common sites of metastasis in SFT patients were reported to be the lung and the liver [12, 14–31]. In addition, there are few case reports of SFTs, mostly arising from the meninges and pleura, presenting with multiple late distant bone metastases that followed the prior evidence of lesions located to the lung and to the liver. To our knowledge there are only two reports of SFTs relapsed with a single late bone metastasis and no extra-skeletal [32, 33]. Notably in both cases primary tumour was located to the meninges.

Our study confirms that isolated bone metastasis can occur in SFT. To note, in our series, in two of four cases the primitive tumour arose from the soft tissue.

Recently also primary SFT arising from the bone have been reported [26]. In case of a single bone lesion consistent with SFT a past or present primary tumour needs always to be ruled out.

In our series, median time from the primary tumour and the appearance of bone relapse was about 3 years, while published cases are reported after a long interval from the primary [32, 33].

All our patients received a local treatment of the bone metastasis with a curative intent. This could be considered an overtreatment as the standard of care of bone metastasis is offered with a palliative intent. Curative surgery was selected in four patients, followed by complementary radiotherapy in two cases (cases 3, 4, Table 1), while radiotherapy was given in two cases. However, it is interesting to note that in two cases with a prolonged follow-up the tumour has not yet relapsed. Yet to be confirmed on a larger prospective series, this suggests that in case of single bone metastasis a local treatment with curative intent may be an option.

In addition, in one of the two cases, the selected treatment was definitive RT alone suggesting that radiation treatment can be an alternative to surgery when morbidity is an issue. No patients received a systemic treatment for the single bone lesion; chemotherapy was given later in two patients who relapsed to multiple sites. SFTs show a low sensitivity to conventional cytotoxic chemotherapy [34, 35]. Recently, systemic therapy has focused on molecularly targeted therapies reporting some activity of antiangiogenics (bevacizumab in combination with temozolomide, sorafenib, sunitinib and pazopanib) [35–37].

There is no consensus on the optimal routine follow-up policy of sarcomas [38]; although SFTs presenting with a single bone metastasis seem to be relatively rare, this series suggests that a bone scan should be included in the staging of SFT patients and in case of bone pain in a patient with a history of SFT.

In one case we observed a bone lesion progressing towards a more aggressive variant of SFT. This underlies once more the limits of the classification available to date [39]. None of our cases showed, at the time of the skeletal progression, a biological shift towards a high-grade dedifferentiated SFT. However, some of the cases showed an initial loss of STAT6 nuclear positivity in the bone metastasis, suggestive for a more aggressive potential. It is now described in the literature that immunohistochemical positivity for STAT6 may be lost in some SFTs while the fusion *NAB2*–*STAT6* is retained [9]. To rule-out SFT diagnosis in these cases the demonstration of translocation is needed.

## Conclusion

This small series in a rare sarcoma subtype suggests that isolated, skeletal metastases are a possible event in both meningeal and extrameningeal SFTs. On this basis bone lesions or symptoms in a patient with a history of SFT should be always investigated. Potentially curative local treatments may be an option, although a larger series is needed to define the best treatment strategy for such patients.

## Abbreviations

SFT: solitary fibrous tumour
CT scan: computerized tomography
MRI scan: magnetic resonance imaging
RT: radiotherapy
CT: chemotherapy
IH: immunohistochemistry
NA: not applicable
M: months
Met: metastasis
Pos: nuclear positivity
*: negativity of isolated neoplastic cells
NED: no evidence of disease
AWD: alive with disease
DOD: died of disease
OS: overall survival
